# LINC00518 Promotes Cell Proliferation by Regulating the Cell Cycle of Lung Adenocarcinoma Through miR-185-3p Targeting MECP2

**DOI:** 10.3389/fonc.2021.646559

**Published:** 2021-04-15

**Authors:** Xu Han, Jixiang Wu, Yajun Zhang, Jianxiang Song, Zhan Shi, Huiwen Chang

**Affiliations:** Department of Thoracic and Cardiovascular Surgery, The Sixth Affiliated Hospital of Nantong University, Yancheng, China

**Keywords:** NSCLC, LINC00518, cell cycle, MECP2, miR-185-3p

## Abstract

Previous studies have shown that long intergenic non-protein coding RNA 00518 (LINC00518) are essential for the cell growth and metastasis of human cancer. However, the role of LINC00518 in lung adenocarcinoma (LUAD) is still unknown. This research put emphasis on the function of LINC00518 on the cell growth of LUAD. The lncRNA, miRNA and mRNA expression were measured by using qRT-PCR. Protein levels were measured by using Western blotting. CCK-8, colony formation assays and transwell assay were performed to evaluate the cell proliferation ability and invasion. Bioinformatic analysis and luciferase reporter assays were chosen to confirm the mechanism of LINC00518 in LUAD. We found that LINC00518 was highly expressed in LUAD specimens and the high-expression was negatively correlated with the overall survival rates. This finding was also proved in the LUAD cell lines. Through a series of *in vitro and in vivo* experiments, we proved that LICN00518 promoted the cell growth of LUAD by regulating the cell cycle. Moreover, LICN00518 upregulated the expression of MECP2 by mutagenesis of miR-185-3p. The results suggested that LICN00518 could be used as a survival indicator and potential therapeutic target for LUAD patients.

## Introduction

Lung cancer still remains the highest rates of morbidity and mortality among all malignancies (1). At present, more than 800,000 new cases of lung cancer are reported annually all over the world, of which 700,000 are fatal (2). Non-small cell lung cancer (NSCLC) is a major subtype of lung cancer, accounting for 85% of lung cancer, and consists of three main subtypes: lung adenocarcinoma (LUAD), lung squamous cell carcinoma (LSCC), and large cell carcinoma (3). In addition, LUAD accounts for the majority of NSCLC. At present, the main effective treatment methods for lung cancer are: surgery, radiotherapy and chemotherapy, immune and targeted therapy (4, 5). Despite these recent advances in the treatment of lung cancer, the 5-year survival rate is still not satisfactory (6). Therefore, early diagnosis and molecular targeted therapy are critical to enhance the prognosis of patients with LUAD.

Long intergenic noncoding RNAs(lincRNAs)have been highlighted as new regulatory molecules characterized by cell-type specific expression and subcellular compartment localization (7). LincRNAs play a critical role in the pathogenesis and development of cancer by interacting with cancer related genes (8, 9). Previous studies have shown that LINC-PINT promotes cell proliferation through EZH2 and predicts poor prognosis in clear cell renal cell carcinoma (10). In addition, Linc-ROR targets FGF2 to regulate HASMC proliferation and migration via sponging miR-195-5p (11). LINC00518 was found to be highly expressed in many human cancers including gallbladder cancer and colorectal cancer and acted as a functional player in tumor cell proliferation, migration and invasion (12, 13). Nevertheless, the specific role of LINC00518 in LUAD needs more complete exploration.

In this manuscript, the authors first discovered that LINC00518 was highly expressed in LUAD tissues and cell line. *In vitro and in vivo* experiments have demonstrated that LINC00518 promoted cell proliferation by regulating the cell cycle of LUAD through miR-185-3p targeting MECP2. The LINC00518/ miR-185-3p/MECP2 axis may have therapeutic potential for lung cancer tumorigenesis and development.

## Materials and Methods

### Patients and Tissue Samples

Since 2019, 42 LUAD patients were selected form The Sixth Affiliated Hospital of Nantong University. This research was approved by the Ethics Committee of Yancheng Third People’s Hospital. Patients who received preoperative radiotherapy or chemotherapy, and those who had a history of other cancers were excluded from the study. All patients voluntarily participate in this study.

### Cell Culture and Transfection

SPCA1, H1299, PC9, A549(LUAD cells), 16HBE (human bronchial epithelial) cells and HEK293T cells were purchased from the Chinese Academy of Sciences (Shanghai, China) and were cultured in DMEM medium (Gibco, NY, USA), with 10% fetal bovine serum (Gibco, NY, USA), and placed in a 37 °C incubator containing 5% CO2. LINC00518 siRNA, LINC00518 overexpression plasmid, miR-185-3p mimics, miR-185-3p inhibitor, NC were purchased from Genechem (Shanghai, China). Transitory transfection was performed using lipofectamine 3000 (Invitrogen, USA) in accordance with the manufacturer’s instructions.

### Isolation of Total RNA and Quantitative Real-Time Polymerase Chain Reaction (qRT-PCR)

Total RNA was isolated from LUAD tissues or cells using Trizol reagent (Invitrogen, USA). Reverse transcription was performed according to the instructions of Prime Script RT reagent (Takara, Kusatsu, Japan). QRT-PCR was conducted by using SYBR Green Master Mix I (Takara, Kusatsu, Japan) on an ABI 7500 Fast Real Time PCR System (ABI, CA, USA). miRNA samples were calibrated with U6, lncRNA and mRNA samples were calibrated with GAPDH. Relative quantification was performed by using the 2-ΔΔCT method. For miRNAs, primer probes were designed and synthesized by Gemma (Shanghai, China). For mRNA, Primer 5.0 (Gemma, Shanghai, China) was used to design the gene primers. All the oligonucleotide sequences used in the experiment are shown in **Table 1**.

**Table 1:** The oligonucleotides used in this study.

Name^a^	Sequence (5’- > 3’)
LINC00518 F	GTGAAAATCTGGCTACTCGTCCC
LINC00518 R	CTGACTTTTGCCACAGACTCCTG
miR-185-3p F	GATCACACTCTTGTGGTAGTTGC
miR-185-3p R	CTCTTCCTTGCTCGTTGTTGGTAT
miR-185-3p mimic	AGGGGCUGGCUUUCCUCUGGUC
miR-185-3p mimic-NC	UUCUCCGAACGUGUCACGUTT
miR-185-3p inhibitor	GACCAGAGGAAAGCCAGCCCCU
miR-185-3p inhibitor-NC	CAGUACUUUUGUGUAGUACAA
MECP2 F	GCCGAGAGCTATGGACAGCA
MECP2 R	CCAACCTCAGACAGGTTTCCAG
GAPDH F	GTCAACGGATTTGGTCTGTATT
GAPDH R	AGTCTTCTGGGTGGCAGTGAT

a F: forward primer, R: reverse primer.

### RNA Binding Protein Immunoprecipitation (RIP)

The EZMagna RIP kit (Merck, Darmstadt, Germany) was used to perform RIP analysis according to the manufacturer's protocol. First, the RIP lysis buffer was used to lyse HEK293T cells. And then the lysate products were Incubate with magnetic beads at 4°C for 6 hours that were pre-conjugated with anti-Ago2 or anti-IgG antibody. Protease K was used to obtain purified RNA by eliminating proteins. Finally, the mRNA level was determined by qRT-PCR.

### Colony Formation Assay

The LUAD cells of each group in the logarithmic growth phase were inoculated on a 35mm petri dish (Corning, NY, USA) with 10 mL of 37°C pre-warmed culture medium and cultured for 2 weeks. Then, the petri dish was stained with crystal violet staining solution (Beyotime, Shanghai, China), and colonies with ≥10 cells were counted under the microscope with low magnification. After all preparations, three independent experiments were carried out.

### Cell Counting Kit-8 (CCK-8) Analysis

Stably transfected LUAD cells were incubated in 96-well plates (Corning, NY, USA) and then stained with 10 μl of CCK-8 solution (Dojindo, Tokyo, Japan) for 2 hours at 37°C. Each absorbance (A450) was measured at 450 nm using a spectrophotometer. Each sample was run in triplicate.

### Flow Cytometry

First collect the cells in each group, centrifuge and suspend them in 0.3ml of PBS containing 10% calf serum, then add 0.7ml of absolute ethanol to fix the cells and place them at -20°C for 24 hours. Discard the supernatant and resuspend the cells with 1ml PBS, and then centrifuge to wash the cells once. After discarding the supernatant, 100μL RNase A was added to the cells, and then placed at 37°C for 30 minutes. Then, 400 μL of propidium iodide (PI) was added to the cells and incubated in the dark for 10 minutes. Finally, the cell cycle was detected by flow cytometry (BD Biosciences, Detroit, USA).

### Luciferase Report Analysis

The 3'-UTR sequence of MECP2 and LINC00518 containing the binding site of miR-185-3p were inserted into the promoter vector (Promega, Madison, USA). HEK293T cells were seeded in six-well plates (Corning, NY, USA) and transfected with related oligonucleotides and luciferase reporter plasmids by the Lipofectamine 3000 (Invitrogen), and transfected cells were obtained after 48 hours. Relative luciferase activity was evaluated using a luciferase assay kit (Promega).

### Western Blotting

Total protein was isolated from cells by using RIPA Lysis Buffer (Beyotime, Shanghai, China). BCA Protein Assay Kit was used to evaluate the protein concentration. The extracted protein was separated by 10% SDS-PAGE and transferred to a polyvinylidene fluoride (PVDF) membrane (Bio-Rad, CA, USA). After blocking with 5% nonfat dry milk for 1 hours, the membrane was cultured with specific primary antibody at 4 °C overnight. The membrane was later cultured with HRP-conjugated secondary antibody (1:1000) for 2 hours at room temperature. An enhanced chemiluminescence (ECL, Rockford, USA) detection system was applied to detect protein blots.

### 5-Ethynyl-2′-deoxyuridine (Edu) assay

Cells are seeded into 96-well plates (5×104 cells/well). Add DMEM (10% FBS) containing 100uLEdU (50μmol/L) to each well and incubate for 2 hours. After washing the cells with PBS, fix them in 4% paraformaldehyde for 15 minutes. 1x ApolloR reaction mixture (400μL) was add to react with EdU (RiboBio, China) at room temperature for 30 minutes, and infiltrate with 0.5% TritonX-100 for 10 minutes. Then the cells was washed with PBS, and incubated with Hoechest33342 (500μL) at room temperature for 30 minutes. After washing the cells with PBS, observe the cells through a fluorescence microscope and take pictures. The mean number cells of each sample in the three fields were counted under the microscope.

### Animal experiments

Animal experiments were approved by the Ethics Committee of Yancheng Third People’s Hospital. si-LINC0051 cells transfected with lentivirus were injected into 4-week-old nude mice (five mice per group). Tumor volume were measured every 4 days. 24 days after Injecting, the mice were euthanized, and then the tumors were resected for weighting. PerkinElmer IVIS Spectrum (Xenogen, CA) was used for in vivo imaging.

### Immunohistochemistry (IHC)

All specimens were fixed with formalin and embed them in paraffin. After the specimen was sectioned, it was deparaffinized with xylene and then hydrated with alcohol. Then the section of specimens were incubated with primary antibodies Ki-67 and P-Akt (Abcam, USA) at 4°C overnight, and then HRP-conjugated secondary antibodies were added and incubated at 4°C for 1 hour. Subsequently, DAB developer was added for dyeing and counterstained with hematoxylin. The images were taken by fluorescent microscope. Three visual fields were selected for each group.

### Statistical Analysis

All experimental data were obtained using SPSS 23.0 and GraphPad software 7.0. The P-values were analyzed by performing Student’s t-test, one-way ANOVA and Spearman’s test. P < 0.05 was considered statistically significant.

## Results

### LINC00518 Was Over-Expressed in LUAD Tissues and Cell Lines

**Figure 1:**
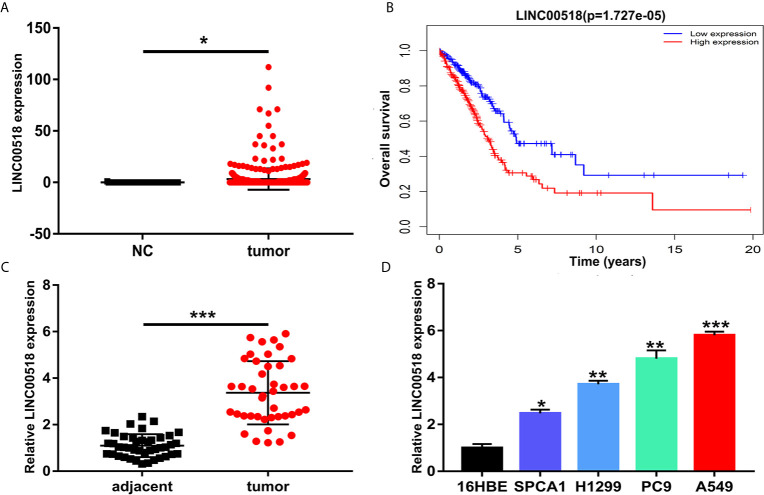
LINC00518 was over-expressed in LUAD tissues and cell lines. **(A)** The expression level of LINC00518 in LUAD tumor tissues and normal tissues form the TCGA database. **(B)** Survival rate of patients with high and low LINC00518 expression. **(C)** LINC00518 expression in LUAD tumor tissue compared with normal adjacent tissue. **(D)** Expression level of LINC00518 in human bronchi Epithelial Cells (16HBE) and LUAD cell lines(SPCA1, H1299, PC9, A549). Data are represented as mean ± SD. (*P < 0.05, **P < 0.01, ***P < 0.001).

Through the TCGA database, we found that compared with normal lung tissues, the expression of Linc00518 in cancer tissues of LUAD patients was significantly increased (**Figure 1A**). In addition, data from the TCGA dataset showed that patients with high expression of LINC00518 had poorer overall survival (**Figure 1B**). Due to the differential expression of LINC00518 in the database, we used qRT-PCR to detect the expression of LINC00518 in tumor tissues and adjacent tissues in 42 pairs of LUAD samples. The data showed that LINC00518 expression was significantly higher in LUAD tumor tissues compare with normal lung tissue (**Figure 1C**). In addition, the expression of LINC00518 in a series of human LUAD cell lines, were further verified this finding. The expression of LINC00518 in LUAD cell lines (SPCA1, H1299, PC9, A549) was significantly higher than that of 16HBE (Human bronchial epithelial cells) (**Figure 1D**). As shown in **Table 2**, in clinical samples, patients with larger tumor size and late pathological stage (TNM stages) have higher LINC00518 levels. Above all, LINC00518 may play an important role in the proliferation of LUAD cells.

**Table 2:** The Correlation between LINC00518 Expression and Clinicopathological Features in 42 LUAD Patients.

Features	All cases	LINC00518 expression		
	Total	Low	High	P value
**Total number**	42	20	22	
**Age**				
>50	27	12	15	0.580
≤50	15	8	7	
**Gender**				
male	18	9	9	0.455
female	24	11	13	
**Lymph node metastasis**				
With	19	7	12	0.204
Without	23	13	10	
**Tumor size (cm)**				
>5	17	3	14	0.001**
≤5	25	17	8	
**TNM stage**				
I+II	20	13	7	0.032*
III+IV	22	7	15	

*P < 0.05, **p < 0.01.

### LINC00518 Promotes LUAD Cell Proliferation

**Figure 2:**
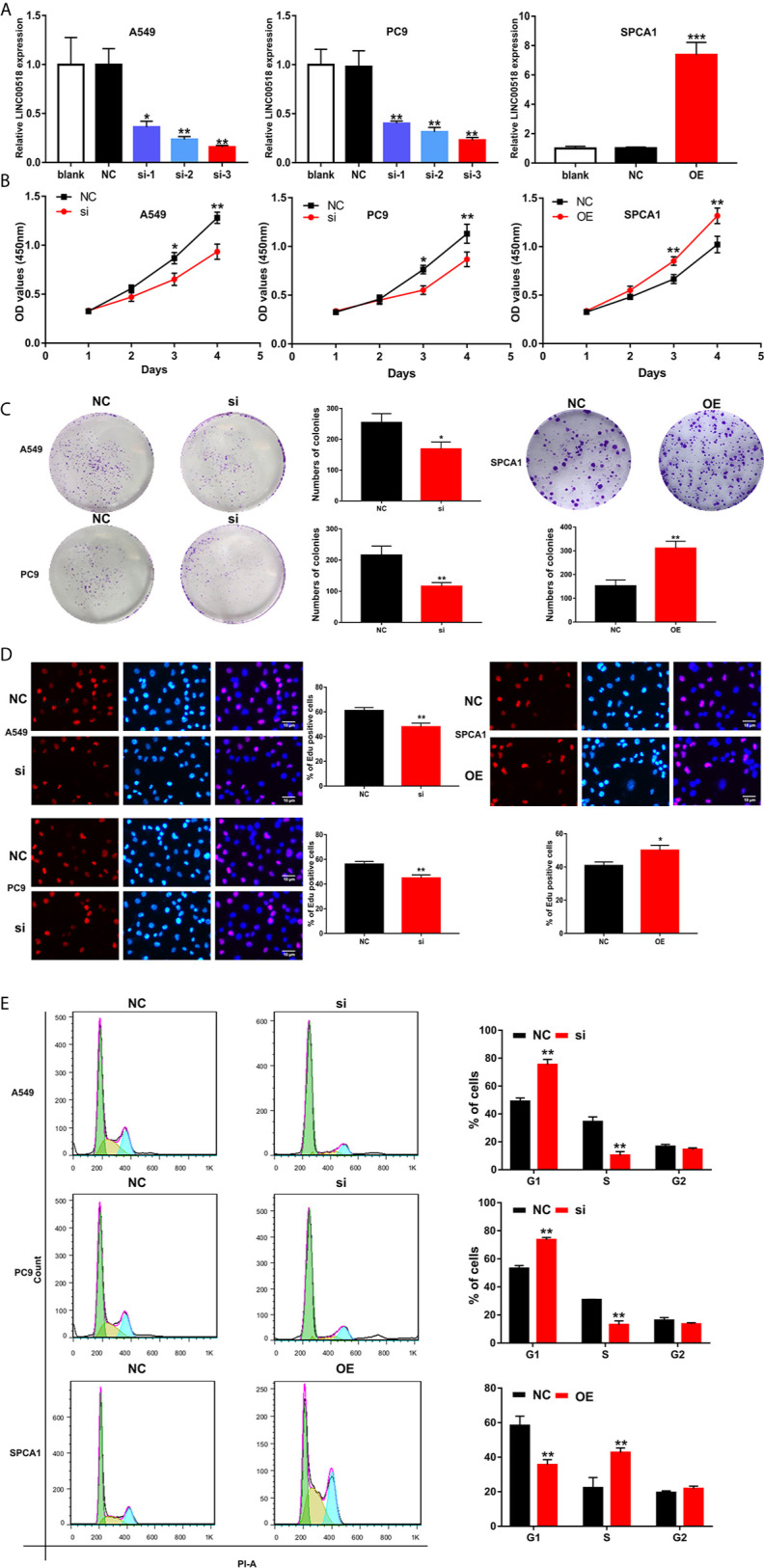
LINC00518 affects LUAD cell proliferation by regulating the cell cycle. **(A)** A549 and PC9 cells were transfected with si-LINC00518 respectively, while SPCA1 cells were transfected with pcDNA-LINC00518. The results were validated by qRT-PCR.NC and blank were used as controls. **(B)** CCK-8 assay was applied to detect the proliferation of A549,PC9 and SPCA1 cells transfected with si-LINC00518 or NC. **(C, D)** Colony formation analysis and Edu assays in A549, PC9 and SPCA1 cells transfected with si-LINC00518,pcDNA-LINC00518 or NC. **(E)** Cell cycle distributions were analyzed in A549, PC9 and SPCA1 cells, respectively by Flow Cytometry. All data are expressed as mean ± SD of three independent experiments. *P < 0.05, **P < 0.01, ***P < 0.001.

In order to study the role of LINC00518 in LUAD cells, we performed RNA interference (small interfering RNA (siRNA)) technology to transfect A549 and PC9 cells respectively, and upregulate LINC00518 by transfected pcDNA-LINC00518 in SPCA1 as overexpression group. Through qRT-PCR analysis, we found that the expression of LINC00518 in A549 and PC9 cells transfected with si-LINC00518 was significantly inhibited, while the expression was increased significantly by transfecting with pcDNA-LINC00518 in SPCA1 cells (**Figure 2A**). Through the CCK-8 assay, compared with the NC group, the cell proliferation of the si-LINC00518 group was significantly reduced, while enhanced LINC00511 expression promoted the proliferation of SPCA1 cells. (**Figure 2B**). The results indicated that down-regulate the expression of LINC00518 can inhibit the proliferation of LUAD cells. The colony formation experiments also showed that the number of colonies formed by A549 and PC9 cells in the si-LINC00518 group were significantly less than that in the NC group, while enhanced LINC00511 expression produced the opposite effect (**Figure 2C**). Similarly, Edu assays showed the same results that LINC00518 affect LUAD cell proliferation (**Figure 2D**). Through flow cytometry analysis, we explored the effect of LINC00518 on the cell cycle of LUAD. As shown in **Figure 2E**, the down-regulation of LINC00518 caused an increase in the proportion of G1 in A549 and PC9 cells, while the proportion of cells in the S phase decreased. These findings indicated that the expression of LINC00518 accelerates the proliferation of LUAD cells by extending the proportion of S phase and reducing the proportion of G1 phase. In addition, we measured the protein level of p-Akt, P-Erk and cyclin D1 in the LUAD cell lines by Western blotting (**Figure S1**). By inhibiting the LINC00518 expression, the protein levels of proliferation-associated protein (p-Akt, P-Erk and cyclin D1) were significantly reduced. The opposite result was obtained after overexpressing LINC00518. Through the above series of in vitro experiments, we confirmed that the overexpression of LINC00518 can promote the proliferation of LUAD cells.

### Knockdown of LINC00518 Inhibits LUAD Tumor Growth *In Vivo*

**Figure 3:**
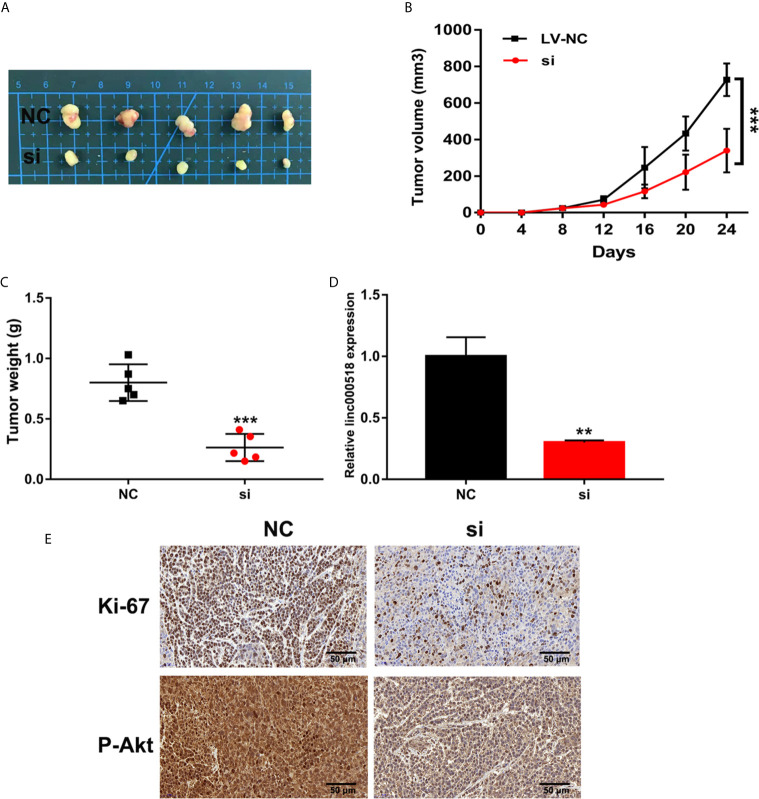
Knockdown of LINC00518 inhibits LUAD tumor growth in vivo. **(A)** Comparison of the effects of transfection with si-LINC00518 or NC on tumor growth in nude mouse models. **(B, C)** Analysis of tumor size and weight in different groups. **(D)** LINC00518 expression changes were identified by qRT-PCR. **(E)** The expression of Ki-67 and P-Akt were detected by IHC of sections from the xenografts (Scale bar, 50 μm). *P < 0.05, **P < 0.01, ***P < 0.001.

We further evaluated the effect of LINC00518 on the growth of LUAD tumors in vivo by inoculating A549 cells transfected with si-LINC00518 into nude mice. The results showed that the low expression of LINC00518 can significantly reduce tumor growth *in vivo* (**Figures 3A-C**). At the same time, the LINC-00518 mRNA level of tumor tissues of nude mice which inoculated with A549 cells transfected with si-LINC00518 was significantly decreased (**Figure 3D**). The results of IHC staining showed that the expression of Ki-67 and p-Akt is consistent with the results of LINC00518 promoting the proliferation of LUAD cells (**Figure 3E**). These results indicated that LINC00518 promotes tumor formation *in vivo*.

### LINC00518 Serves As Competing Endogenous RNA for miR-185-3p in LUAD

**Figure 4:**
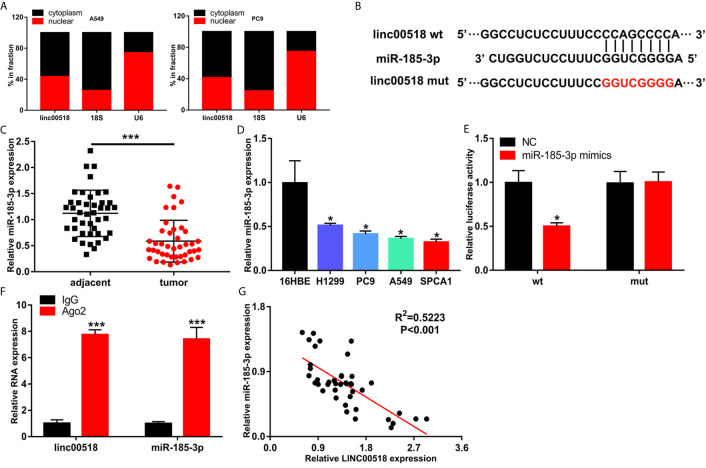
LINC00518 serves as competing endogenous RNA for miR-185-3p in LUAD. **(A)** The subcellular position of LINC00518 on the cytoplasm or nucleus were analyzed by qRT-PCR. 18S and U6 acted as the cytoplasm and nuclear control. **(B)** The putative binding site of miR-185-3p on LINC00518, and LINC00518 MUT. **(C)** The expression level of miR-185-3p in LUAD tissues and adjacent tissues were detected by qRT-PCR. **(D)** The expression levels of miR-185-3p in H1299, PC9, A549, SPCA1 and 16HBE were detected by qRT-PCR. **(E)** Luciferase assay of HEK293T cells transfected with LINC00518 WT or LINC00518 MUT reporter together with miR-185-3p or NC. **(F)** RIP assay was used to further verify the direct interaction between miR-185-3p and LINC00518. **(G)** A negative correlation was found between the mRNA level of LINC00518 and miR-185-3p in 42 LUAD tissues. Data are represented as mean ± SD. (*P < 0.05, **P < 0.01, ***P < 0.001).

Previous studies have shown that LINC00518 plays a role in tumors through the ceRNA mechanism. We used Starbase to predict the LINC00518/miRNA interaction. Finally, we selected miR-185-3p as the research object. QRT-PCR nuclear and cytoplasmic fractions showed that LINC00518 was mainly located in the cytoplasm of LUAD cells (**Figure 4A**). This indicates that LINC00518 may have an effect on post-transcriptional epigenetic regulation. Next, we confirmed that both wild-type and mutant-type LINC00518 have multiple binding sites with downstream miR-185-3p (**Figure 4B**). We also found that miR-185-3p expression was decreased in 42 paired LUAD tumor tissues and LUAD cell lines (**Figures 4C, D**). In the luciferase reporter assays, we found that the luciferase activity of the LINC00518-WT was significantly reduced by transfecting with miR-185-3p mimic (**Figure 4E**). The results of the RIP assay further verify the direct interaction between miR-185-3p and LINC00518. Compared with IgG antibody, the relative RNA expression levels of miR-185-3p and LINC00518 were significantly increased in the immunoprecipitation formed by the Ago2 antibody (**Figure 4F**). In addition, we also found that the mRNA expression levels of LINC00518 was negatively correlated with that of miR-185-3p by qRT-PCR (**Figure 4G**). All results indicated that LINC00518 may act as the ceRNA for miR-185-3p in LUAD.

### LINC00518 Regulate LUAD Cell Proliferation and Cell Cycle by Inhibiting miR-185-3p Expression

**Figure 5:**
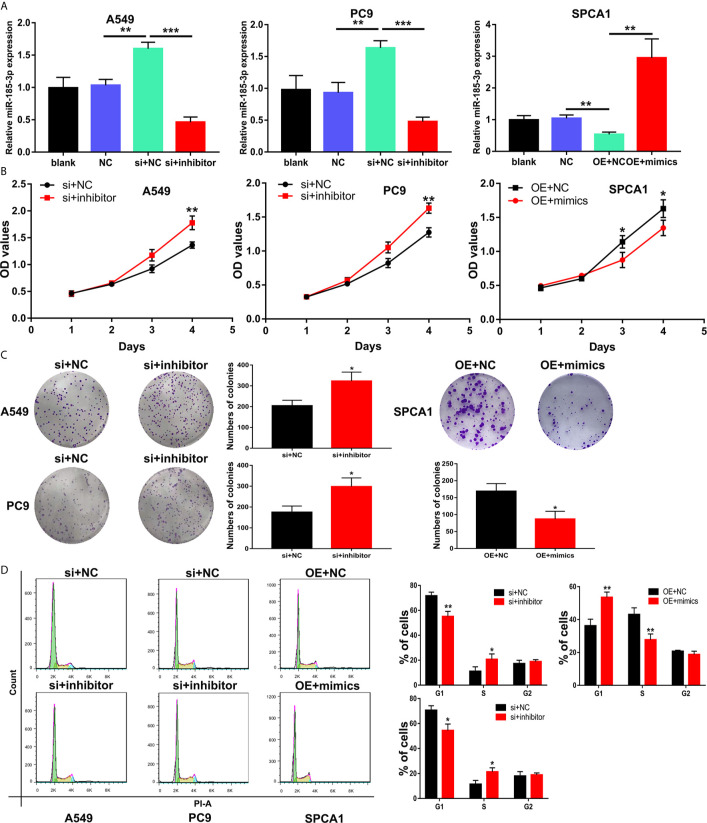
LINC00518 regulate LUAD cell proliferation and cell cycle by inhibiting the miR-185-3p expression. **(A)** The expression levels of miR-185-3p in LUAD cells following transfection with NC, LINC00518 siRNA, LINC00518 siRNA plus miR-185-3p inhibitor, pcDNA-LINC00518 or pcDNA-LINC00518 plus miR-185-3p mimics. **(B, C)** By CCK-8 assay and colony formation assays were used to detect the cell proliferation ability after transfecting A549, PC9 and SPCA1 cells respectively. **(D)** Cell cycle distributions were analyzed in transfected LUAD cells by Flow Cytometry. *P < 0.05, **P < 0.01, ***P < 0.001. Data are represented as mean ± SD. (*indicates P < 0.05, **P < 0.01).

Transfection of si-LINC00518 in A549 and PC9 cells can significantly upregulate the expression of miR-185-3p. While transfecting with miR-185-3p inhibitors, the promotion effect of si-LINC00518 on miR-185-3p expression can be significantly reversed. On the contrary, miR-185-3p expression was down-regulated by overexpressing LINC00518 in SPCA1 cells (**Figure 5A**). CCK-8 assay and colony formation assays showed that knockdown of LINC00518 inhibited the proliferation of LUAD cells, and this inhibition was rescued by co-transfection with miR-185-3p inhibitor (**Figures 5B, C**). By flow cytometry analysis, we found that si-LINC00518 co-transfected with miR-185-3p inhibitor can significantly decrease the ratio of G1 and increase the ratio of S-phase cells in A549 and PC9 cells (**Figure 5D**). All these results demonstrated that LINC00518 promoted LUAD cell proliferation and regulate cell cycle by down-regulating miR-185-3p expression.

### MECP2 Is a Target of miR-185-3p Regulated by LINC00518 in LUAD Cells

**Figure 6:**
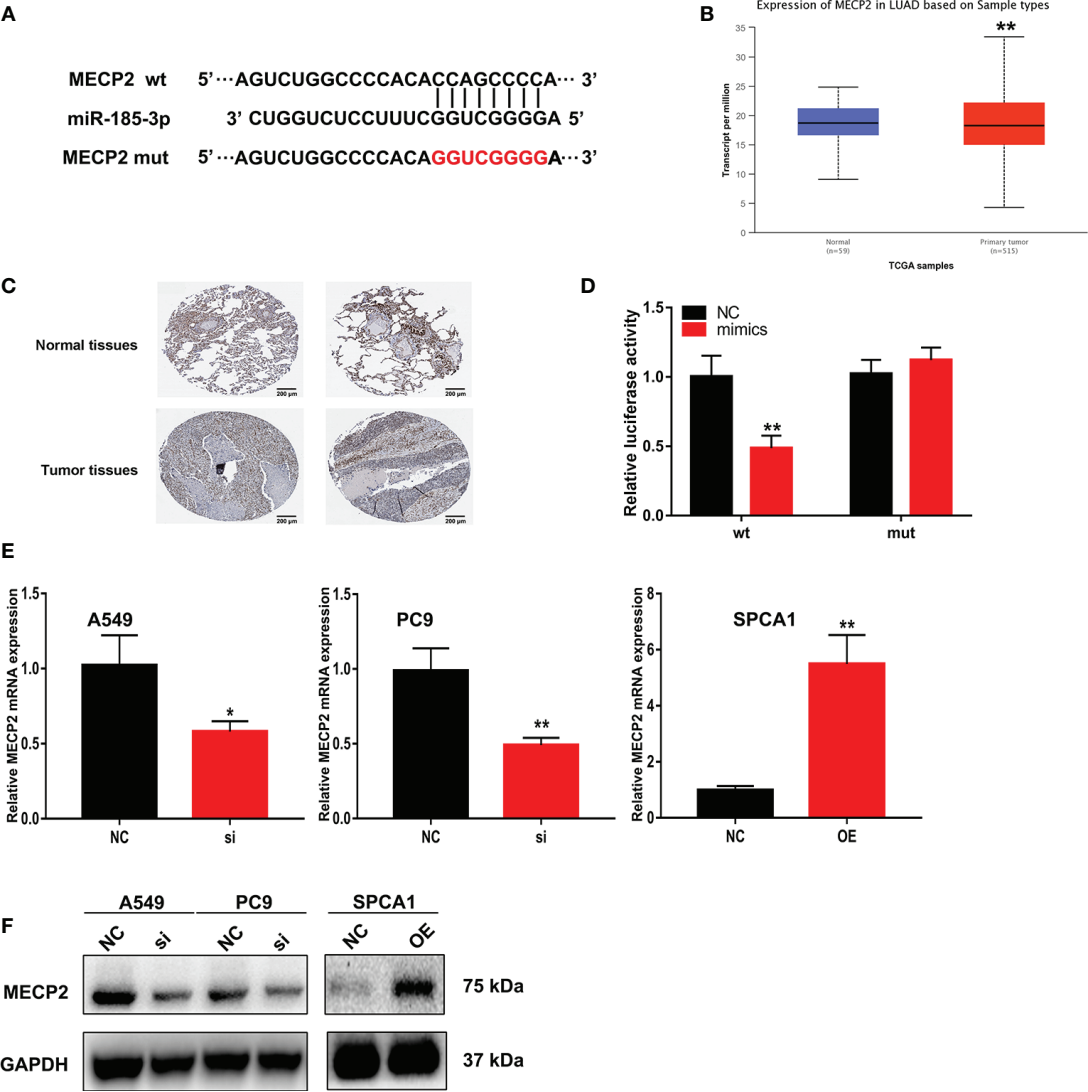
MECP2 is a target of miR-185-3p regulated by LINC-00518 in LUAD cells. **(A)** The binding sites of miR-185-3p on the 3′-UTR of MECP2. **(B)** Relative expression of MRCP2 in LUAD tissues compared with adjacent normal tissues from the TCGA database. **(C)** The expression of MECP2 were detected by IHC from the HPA database. **(D)** Luciferase assay of LUAD cells transfected with MECP2-WT or MECP2-MUT reporter together with miR-185-3p or NC. **(E, F)** Relative mRNA and protein levels of MECP2 in A549, PC9 and SPCA1 cells transfected with NC, si-LINC00518 or pcDNA-LINC00518. Data are represented as mean ± SD. (*indicates P < 0.05, **P < 0.01).

Starbase were also used to predict the putative targets of miR-185-3p. The 3’-UTR of MECP2 has the same binding sites that LINC00518 combined with miR-185-3p (**Figure 6A**). By searching the TCGA database, we found that MECP2 was highly expressed in LUAD samples (**Figure 6B**). The expression of MECP2 detected by IHC from the HPA database demonstrated the consistent results (**Figure 6C**). The luciferase activity of the MECP2-WT was significantly reduced by transfecting with miR-185-3p mimic, but not that of the mutant plasmid (**Figure 6D**). In addition, through qRT-PCR and West blotting, relative mRNA and protein levels of MECP2 were obviously reduced by transfecting with si-LINC00518 (**Figures 6E, F**). These results confirmed that MECP2 is a target of miR-185-3p and LINC00518 regulate the expression of MECP2.

## Discussion

Among all malignant tumors, the incidence and mortality of lung cancer are still at the forefront. Although immunization and targeted therapy have achieved satisfactory results in the diagnosis and treatment of lung cancer, more effective target genes are still needed for early diagnosis and efficacy prediction of lung cancer.

More and more studies have shown that LncRNA plays an important role in the biological process of tumors, such as cell proliferation, differentiation, migration and apoptosis (14, 15). Previous research has shown that Linc-DYNC2H1-4 promotes EMT and CSC phenotypes by acting as a sponge of miR-145 in pancreatic cancer cells (16). Chen et.al reported that Linc-RoR can promote proliferation, migration, and invasion via the Hippo/YAP pathway in pancreatic cancer cells (17).In this study, we investigated the expression pattern and clinical significance of LINC00518 in LUAD tissues by searching the microarray data set published in the TCGA database, and found that the expression of LINC00518 in LUAD tissues and cell lines were significantly up-regulated and the increased LINC00518 expression may be related to a poor survival rate. Besides, by detecting our 42 LUAD samples, we found that the high expression of LINC00518 is significantly related to the advanced TMN stage and lymph node metastasis of LUAD tumors, which indicates that LINC00518 is related to the development of LUAD tumors. Further, We found that knocking down LINC00518 significantly inhibited LUAD cell proliferation and tumor growth in vivo. LINC00518 can also accelerate the proliferation of LUAD cells by extending the proportion of S phase and reducing the proportion of G1 phase. These results indicate that LINC00518 may be an oncogene of LUAD and can be used as an independent potential prognostic biomarker for LUAD patients.

Many studies have proven that lncRNA can exert its function by regulating the expression of miRNA (18–20). For instance, it has been reported that LINC-smad7 promotes myoblast differentiation and muscle regeneration via sponging miR-125b (21).Hao et.al found that LINC-PINT suppresses tumor cell proliferation, migration and invasion through targeting miR-374a-5p in ovarian cancer (22).Previous study has proved that LINC00518 serves as a sponge for the miR-204-5p in melanoma (12). According to the subsequent bioinformatics analyses and luciferase reporter assays, we predicted that the LINC00518 may have a strong interaction with miR-185-3p.In this study, we first proved that miR-185-3p was down-regulated in LUAD tumor tissues and cell lines. We also found that the luciferase activity of the LINC00518-WT was significantly reduced by transfecting with miR-185-3p mimic, and the expression of miR-185-3p is negatively correlated with the expression of LINC00518 in LUAD tissues. Through a series of functional experiments, we found that the effect of LINC00518 in promoting the proliferation of LUAD cells can be reversed by transfection of miR-185-3p mimics. All these results demonstrate that LINC00518 promotes LUAD cell proliferation by down-regulating miR-185-3p expression.

Many studies have shown that miRNAs function by inhibiting the expression of target mRNA. There are reports that MECP2 promotes breast cancer cell proliferation and cell cycle progression and inhibits cell apoptosis (23, 24). MECP2 was also reported as a target of miR-638, facilitated gastric cancer cell proliferation and may serve as a potential target for gastric cancer therapy (25). In our study, MECP2 was selected as a direct target of miR-185-3p through bioinformatics analyses and luciferase reporter assays. We found that the MECP2 expression was up-regulated in LUAD tumor tissues. Through a series of in vitro experiments, we proved that LINC00518 expression has a positive correlation with the expression of MECP2. These findings demonstrated that MECP2 is a target of miR-185-3p regulated by LINC00518 in LUAD cells.

In conclusion, our results prove that LINC00518 promotes cell proliferation and tumor growth by regulating the cell cycle of LUAD through miR-185-3p targeting MECP2 in vitro. In addition, over-expression of LINC00518 is related to the poor prognosis, advanced TMN stages and tumor size of LUAD patients. These data highlight the importance of LINC00518 in the progression of LUAD, indicating that LINC00518 may be a key predictor of LUAD poor prognosis and a potential therapeutic target.

## Data Availability Statement

The datasets presented in this study can be found in online repositories. The names of the repository/repositories and accession numbers can be found in the article/**Supplementary Material**.

## Ethics Statement

The studies involving human participants were reviewed and approved by the Ethics Committee of Yancheng Third People`s Hospital. The patients/participants provided their written informed consent to participate in this study. Written informed consent was obtained from the individuals for the publication of any potentially identifiable images or data included in this article.

## Author Contributions

YZ and JS contributed to the construction of the subject. ZS and HC are responsible for the collection of samples. XH and JW contributed to data analysis and interpretation. XH is responsible for writing, reviewing and/or revising the manuscript. All authors contributed to the article and approved the submitted version.

## Conflict of Interest

The authors declare that the research was conducted in the absence of any commercial or financial relationships that could be construed as a potential conflict of interest.

## Supplementary Material

Supplementary Figure 1 The protein levels of p-Akt, p-Erk and cyclin D1 in A549, PC9, SPCA1 cell lines transfected with si-LINC00518 or NC were determined by Western blots separately. GAPDH was used as a control.
