# Supplementary figures and images for: LINC00518 Promotes Cell Proliferation by Regulating the Cell Cycle of Lung Adenocarcinoma Through miR-185-3p Targeting MECP2
Source: Front Oncol. 2021 Apr 15;11:646559. doi: 10.3389/fonc.2021.646559 (PMC8081883; doi:10.3389/fonc.2021.646559)

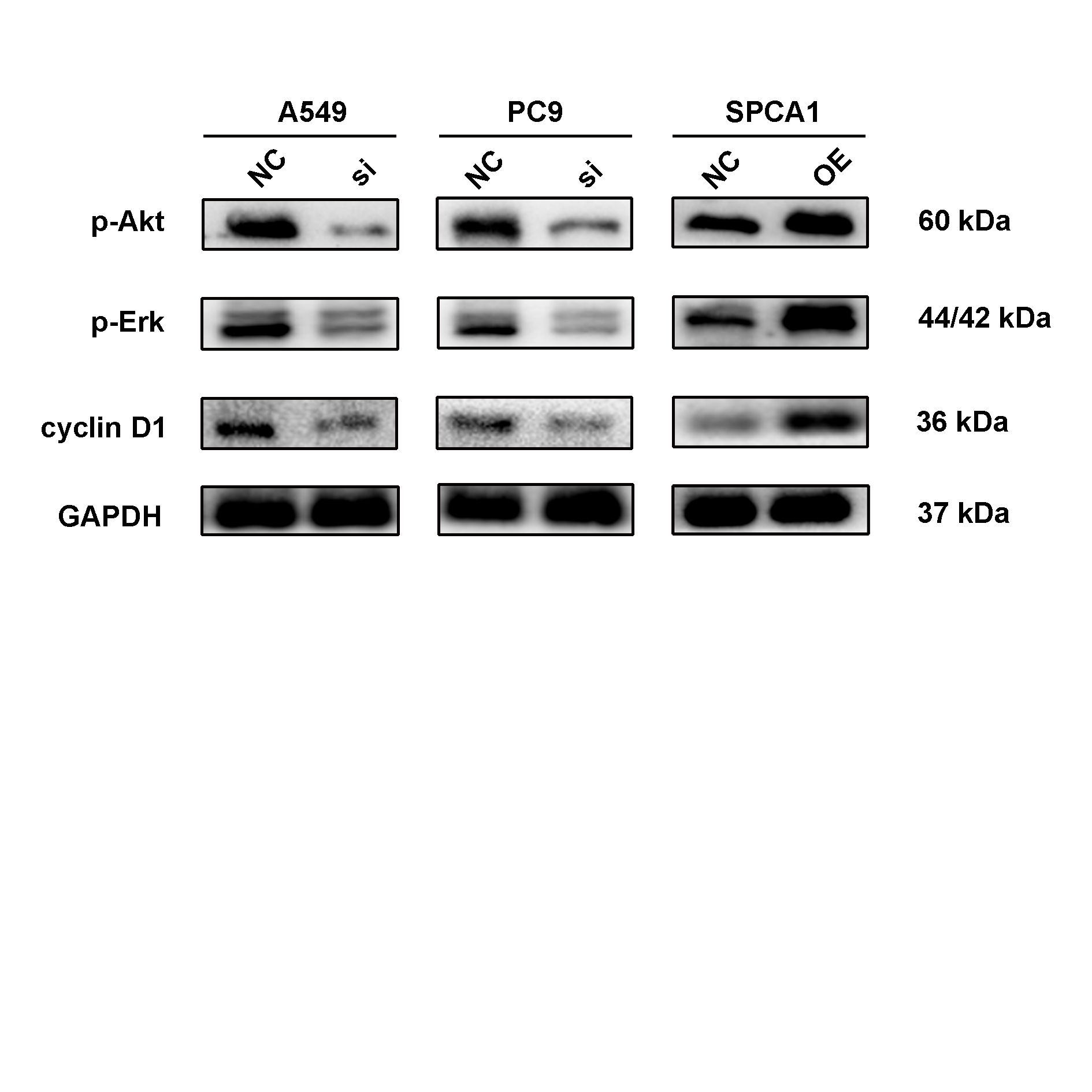

Supplement: Supplementary Figure 1 — The protein levels of p-Akt, p-Erk and cyclin D1 in A549, PC9, SPCA1 cell lines transfected with si-LINC00518 or NC were determined by Western blots separately. GAPDH was used as a control. [file Image_1.TIF]
